# Yield and nitrogen use efficiency in optimized oat-common vetch mixtures on the Qinghai–Tibet plateau

**DOI:** 10.3389/fpls.2026.1782125

**Published:** 2026-04-27

**Authors:** Mingfang Bao, Zhaomin Wang, Meng Yan, Yongchao Zhang, Yan Qin

**Affiliations:** 1 State Key Laboratory of Plateau Ecology and Agriculture, Qinghai University, Xining, China; 2 College of Pastoral Agriculture Science and Technology, Lanzhou University, Lanzhou, China

**Keywords:** growth characteristics, isotopic nitrogen, land equivalent ratio, mixed sowing, Qinghai-Tibet Plateau

## Abstract

This study investigated the systemic effects of various nitrogen (N) regimes and phosphorus (P) additions on growth traits, N accumulation, and N fixation efficiency in mixed sowing systems of oat (*Avena sativa* L.) and common vetch (*Vicia sativa* L.) on the Qinghai-Tibet Plateau. Using a combination of field and pot experiments with a series of N-P co-application treatments, this study systematically measured the biomass, N accumulation, N fixation efficiency, and land equivalent ratio (LER) of two crop species in a mixed sowing system, and compared the results with monoculture treatments. Results indicated that N-enriched conditions significantly enhance biomass, and N accumulation in both species, with the A1V1 treatment (1:1 ratio) showing the highest aboveground biomass (1164 g·m^-2^) and an optimal Land Equivalent Ratio (LER) of 1.86. Phosphorus application improved N fixation and transfer, while moderate N-P co-application optimized N utilization within the system. Mixed sowing demonstrated superior N fertilizer utilization compared to monoculture, promoting sustainable agriculture by enhancing crop resilience and productivity. Adjustments in N-P addition based on specific soil conditions are essential to maximize benefits and avoid negative impacts on biological N fixation.

## 1 Introduction

Grass and legume forages, as two of the most widely distributed food and forage crops worldwide, exhibit pronounced resource complementarity. In particular, the biological nitrogen(N)-fixing capacity of legumes has made grass-legume mixed sowing systems a highly efficient cultivation model that is widely promoted internationally (Hu et al., 2016). Compared to monocropping, grass-legume mixed sowing system offers advantages such as higher land utilization, lower fertilizer input, greater plant yield and quality, and improved agricultural benefits (Li et al., 2020; Lithourgidis et al., 2011). Research indicates that grass-legume mixed sowing systems offer significant advantages, including increased forage biomass, enhanced soil nutrient use efficiency, suppression of field pests and diseases, and reduced environmental pollution (Chen et al., 2014; Wang et al., 2019; Yang et al., 2019).

In grass-legume mixed sowing systems, the sowing ratio is one of the factors governing both productive performance and ecological benefits (Li et al., 2020; Luo et al., 2023; Wu et al., 2020). Previous studies have shown that an optimal mixing ratio can significantly improve the soil microenvironment, increase soil microbial abundance and activity, and promote the mineralization of soil carbon and N, thereby conferring favorable production and ecological properties on the soil and ultimately increasing yields (Elgersma and Hassink, 1997; Lithourgidis et al., 2006; Qi et al., 2016; Rodriguez et al., 2020; Wu et al., 2022; Yan et al., 2022; Xu et al., 2023). The sowing ratio not only influences the productive performance of the system, but also strongly affects N-use efficiency. The N-fixing capacity of legumes can supply N to grass forages, reducing reliance on external N fertilizers and enhancing N-use efficiency (Zhang et al., 2019; Chen et al., 2023). An appropriate sowing ratio can optimize N allocation and utilization within the system, minimize N losses, and reduce the risk of environmental pollution (Qin et al., 2020).

Oats (*Avena sativa* L.) and common vetch (*Vicia sativa* L.) are highly suitable forage crops for the Qinghai-Tibet Plateau (Boetzl et al., 2023). Although common vetch is capable of fixing atmospheric N, its effects on N dynamics in mixed sowing systems, and its influence on N uptake by oats, remain insufficiently understood (Li et al., 2001; Hu et al., 2016; Luo et al., 2023; Novoa and Loomis, R.S., 1981). In this study, a field experiment was conducted on the Qinghai–Tibetan Plateau in 2021. Multiple oat–common vetch sowing ratios and monocultures were tested under two N levels to evaluate forage yield, land equivalent ratio, and N uptake, and to identify the optimal mixture for local conditions. The optimal mixture was then used in a ^15^N tracer pot experiment to quantify interspecific N transfer under different N and phosphorus (P) levels, and to test whether legume-to-cereal N transfer underlies the yield advantage of the mixture. Together, these two complementary experiments link field-scale agronomic performance with interspecific N transfer processes, providing a mechanistic basis for evaluating oat–common vetch mixtures under the semi-arid, cold conditions of the Qinghai–Tibetan Plateau.

## 2 Materials and methods

### 2.1 Experimental site overview

The experimental site is established at the Perennial Forage Germplasm Resource Nursery in Xihai Town, Haibei Prefecture, Qinghai Province, China (101°45′E, 36°49′N). The site is situated at an elevation of 3150 m and experiences an annual precipitation of 375 mm, an annual evaporation of 1762.8 mm, an average annual temperature of 5.7°C, and an annual sunshine duration of 2762.0 hours. The study area is characterized by high solar radiation, large diurnal temperature variations, a short growing season, and represents a typical plateau climate.

### 2.2 Experimental material and design

The experiment was conducted in 2021, using a completely randomized design with 30 plots and three replicates per treatment, and lasted 143 days. Calcium superphosphate [Ca(H_2_PO_4_)_2_·H_2_O] was applied at 60.0 g·m^-2^ before sowing, and manual weeding was performed twice during the growth period. The experimental materials selected for this study include oats (*Avena sativa* L., cv. ‘Qingyan No. 1’) and common vetch (*Vicia sativa* L., cv. ‘Ximu 324’). The seeds were provided by the Qinghai Academy of Animal Husbandry and Veterinary Sciences.

Referring to Liu et al (Liu et al., 2019), the highest forage yield and nutritive value of monoculture oat on the alpine Qinghai–Tibet Plateau occurred at 20 g N·m^-2^, whereas those of monoculture common vetch occurred at 60 g P·m^-2^. The following N and P levels were therefore used for oat and common vetch in the mixed oat–vetch grassland experiment. Seeding rates followed local recommendations, and five mixed sowing ratio treatments were established as follows:

1. Two N (urea: CO(NH_2_)_2_) conditions were established: LN (no additional urea) and HN (20.0 g·m^-^² of urea);
2. Oat monoculture: seeding rate 22.5 g·m^-2^ (A.M.);
3. Common vetch monoculture: seeding rate 12.0 g·m^-2^ (V.M.);
4. Oats: common vetch 2:1 mixed sowing (A2V1);
5. Oats: common vetch 1:1 mixed sowing (A1V1);
6. Oats: common vetch 1:2 mixed sowing (A1V2).

In addition, based on the above field results, a ^15^N tracer pot experiment was conducted in 2023 to assess yield advantages and N transfer in the 1:1 oat–common vetch mixture (A1V1) under different nutrient regimes. The experiment used a factorial design with two N and one P levels. Nitrogen was applied as urea, and P as calcium superphosphate [Ca(H_2_PO_4_)_2_·H_2_O].

All treatments were grown in plastic pots (30 cm diameter, 25 cm height). In N-fertilized treatments, ^15^N-labeled urea replaced the corresponding amount of unlabeled urea. The fertilizer was thoroughly mixed into the 0–5 cm soil layer to enable precise tracing of N transfer and transformation. Subsequently, the samples will be dried at 110°C to terminate any biological activity and then further dried at 65°C until a constant weight is achieved to determine the dry matter biomass. For isotopic N analysis, the dried and powdered plant samples will be analyzed for ^15^N content using a mass spectrometer (MAT 253).

### 2.3 Sample collection and processing

Five 1-m sampling segments were randomly collected from each plot. Forage were cut at a stubble height of 3–5 cm, and the root systems were excavated. Forage were separated into stems, leaves (including leaves and spikes for oat and leaves and pods for common vetch), and roots, and fresh weight was recorded. In the laboratory, samples were first oven-dried at 105°C for 30 min to deactivate biological activity, and then dried at 65°C to constant weight to determine dry biomass. Nitrogen concentrations in the digested plant samples of each organ were measured using a flow injection analyzer (FIAstar 5000).

### 2.4 Data analysis

The data can be entered and computed using Microsoft Excel (version 16.86). One-way analysis of variance followed by the least significant difference test was performed using SPSS software to assess the significance of differences for each indicator among different fertilizer treatments (*P* < 0.05). Two-way ANOVA was also conducted to evaluate the effects of fertilizer treatments on the measured variables. The plyr package in R was used in combination with the multi-criteria decision-making model TOPSIS (technique for order preference by similarity to an ideal solution) to comprehensively evaluate the different fertilizer treatments. Piecewise structural equation modeling (piecewise SEM) was constructed using the piecewise SEM package in R 4.0.2 (R Development Core Team) to explore the influence pathways and path coefficients of N, P, and their combination on the yield of grass–legume mixtures. Figures were generated using Origin 2024.


$$\begin{array}{c} \text{LER}=\text{LERa}+\text{LERb}\\ =(\text{yield of crop a in the mixture}/\\ \text{yield of crop a in monoculture})\\ +(\text{yield of crop b in the mixture}/\\ \text{yield of crop b in monoculture}); \end{array}$$

$$\begin{array}{c} \text{N accumulation }(\text{g}\cdot {\text{m}}^{-2})\\ =\text{dry matter yield}\times \text{N concentration in dry matter}; \end{array}$$

$$\begin{array}{c} \text{N accumulation }(\text{g}\cdot {\text{m}}^{-2})\\ =\text{dry matteN partial factor productivity }(\text{PFPN},\text{ kg}/\text{kg N})\\ =\text{Crop yield with N fertilizer}/\text{Amount of N fertilizer}; \end{array}$$

$$\begin{array}{c} \text{N agronomy efficiency}(\text{AEN},\text{ kg}/\text{kg N})\\ =\text{Difference in crop yield with and without N}/\\ \text{Amount of N fertilizer}; \end{array}$$

$$\begin{array}{c} \text{N recovery efficiency}(\text{REN},\;\%)\\ =(\text{Difference in N accumulation in aboveground parts }\\ \text{with and without N}/\text{Amount of N fertilizer})\times 100\%; \end{array}$$

$$P_{Ndfa}(\%)=(1-\frac{\delta^{15}N_{fix}-\delta^{15}N_{ref}}{\delta^{15}N_{non-fix}-\delta^{15}N_{ref}})\times 100\%;$$

Where,

δ^15^ N_fix_: The δ^15^ value of common vetch; δ^15^ N_ref_: The δ^15^ value of oat; δ^15^ N_non-fix_: The δ^15^ value of oat in monoculture.


$${\text{W}}_{\text{Ndfa}}=\text{plant total N content}\times P_{\text{Ndfa}};$$

$$\begin{array}{c} P_{Ndfl}(\%)\\ =(1-\frac{\delta^{15}N_{non-fix}-\delta^{15}N_{mix}}{\delta^{15}N_{non-fix}-\delta^{15}N_{fix}})\times 100\%; \end{array}$$

Where,

δ^15^ N_mix_: The δ^15^ value of oat in the mixed pasture; δ^15^ N_non-fix_-δ^15^: The δ^15^ value of oat in the monoculture pasture; δ^15^ N_fix_: The δ^15^ value of common vetch in the mixed pasture.


$${\text{W}}_{\text{Ndfl}}=\text{total N yield of oat in the mixed treatment}\times P_{Ndfl};$$

$$\text{Contribution rate of biological N fixation}(\%)={\text{W}}_{\text{Ndfa}}\text{/total N yield of the grassland}\times 100\%.$$

## 3 Results

### 3.1 Analysis of growth traits in mixed sowing systems of oat and common vetch

**Figure 1:**
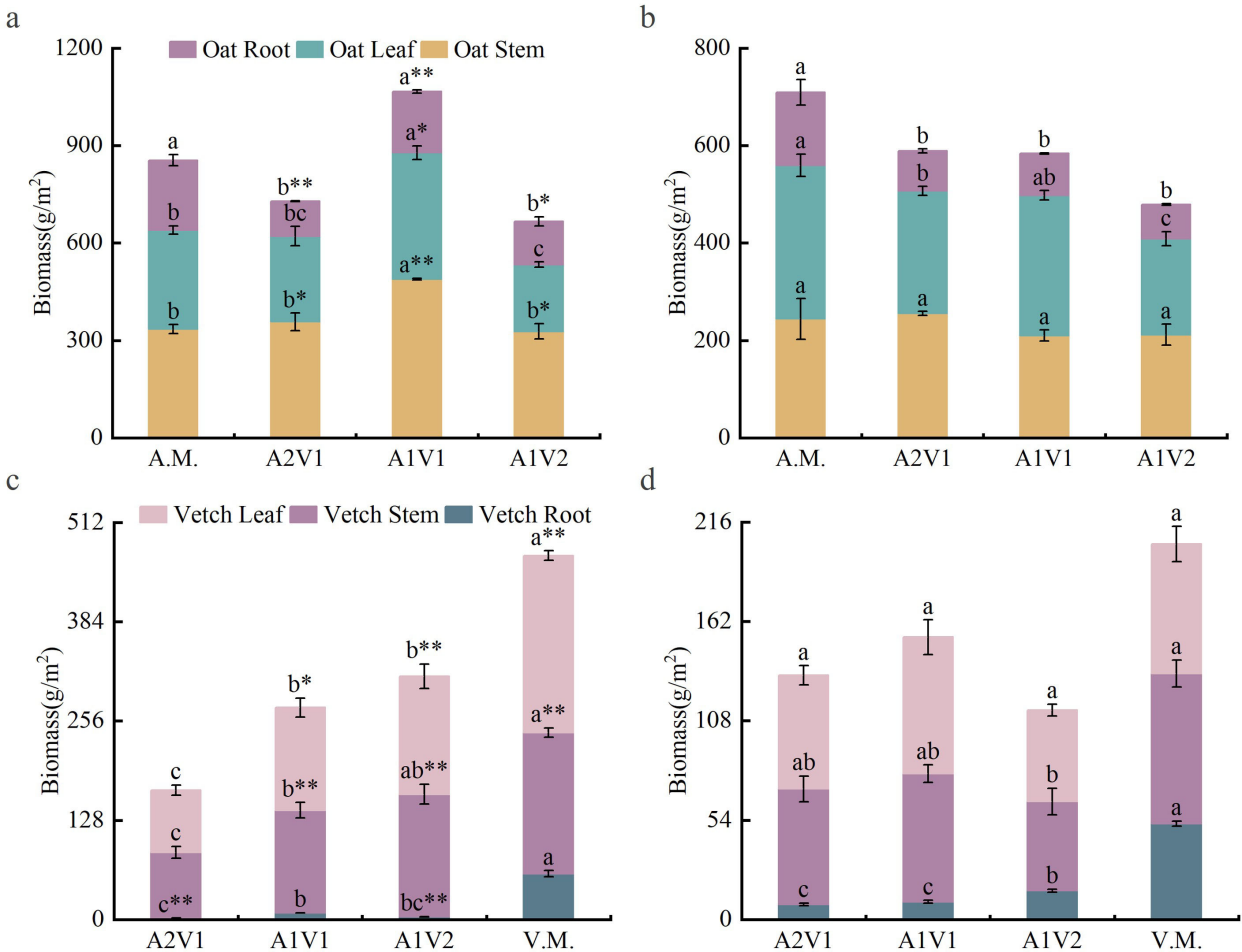
Effects of mixed sowing ratios under two N conditions on biomass allocation of oat and common vetch. **(a)** Biomass allocation of oat under the HN condition. **(b)** Biomass allocation of oat under the LN condition. **(c)** Biomass allocation of common vetch under the HN condition. **(d)** Biomass allocation of common vetch under the LN condition. Different lowercase letters a, b, c indicate significant differences among different sowing ratios within the same N regime (*p* < 0.05). Superscript “*” and “**” indicate significant differences between N regimes for the same mixed sowing treatment at the 0.05 and 0.01 levels, respectively. A.M., A2V1, A1V1, A1V2, and V.M. denote different mixed sowing ratios. HN indicates N-sufficient conditions; LN indicates N-deficient conditions.

Mixed sowing ratios had a significant effect on plant biomass (**Figure 1**, *p* < 0.05). Under the HN conditions (**Figure 1a**), oat stem, leaf, and total aboveground biomass were all highest in the A1V1 treatment, reaching 488.87 g·m^-2^, 389.36 g·m^-2^, and 1066.04 g·m^-2^, respectively, and were lowest in the A1V2 treatment, at 327.38 g·m^-2^, 206.08 g·m^-2^, and 666.28 g·m^-2^, respectively. Oat root biomass was highest in the oat monoculture (214.67 g·m^-2^) and lowest in the A2V1 treatment (107.58 g·m^-2^). Under the LN conditions (**Figure 1b**), oat stem biomass was highest in the A2V1 treatment (255.15 g·m^-2^) and lowest in the A1V1 treatment (209.86 g·m^-2^). However, oat leaf, root, and total biomass showed a declining trend with decreasing oat proportion in the mixture: these variables were highest in the oat monoculture, with values of 315.62 g·m^-2^, 149.24 g·m^-2^, and 708.89 g·m^-2^, respectively, and lowest in the A1V2 treatment, at 196.93 g·m^-2^, 69.96 g·m^-2^, and 478.62 g·m^-2^, respectively. For common vetch, stem, leaf, root, and total biomass under the HN conditions (**Figure 1c**) increased with the increasing legume proportion in the mixture and reached their maximum under monoculture (181.98 g·m^-2^, 228.21 g·m^-2^, 59.06 g·m^-2^, and 469.29 g·m^-2^, respectively). In contrast, under the LN conditions (**Figure 1d**), stem, leaf, and total biomass of common vetch in the A1V2 treatment were the lowest among the three mixed-sowing treatments, with values of 48.47 g·m^-2^, 49.73 g·m^-2^, and 113.80 g·m^-2^, respectively, whereas root biomass was lowest in the A2V1 treatment, at 8.17 g·m^-2^.

**Figure 2:**
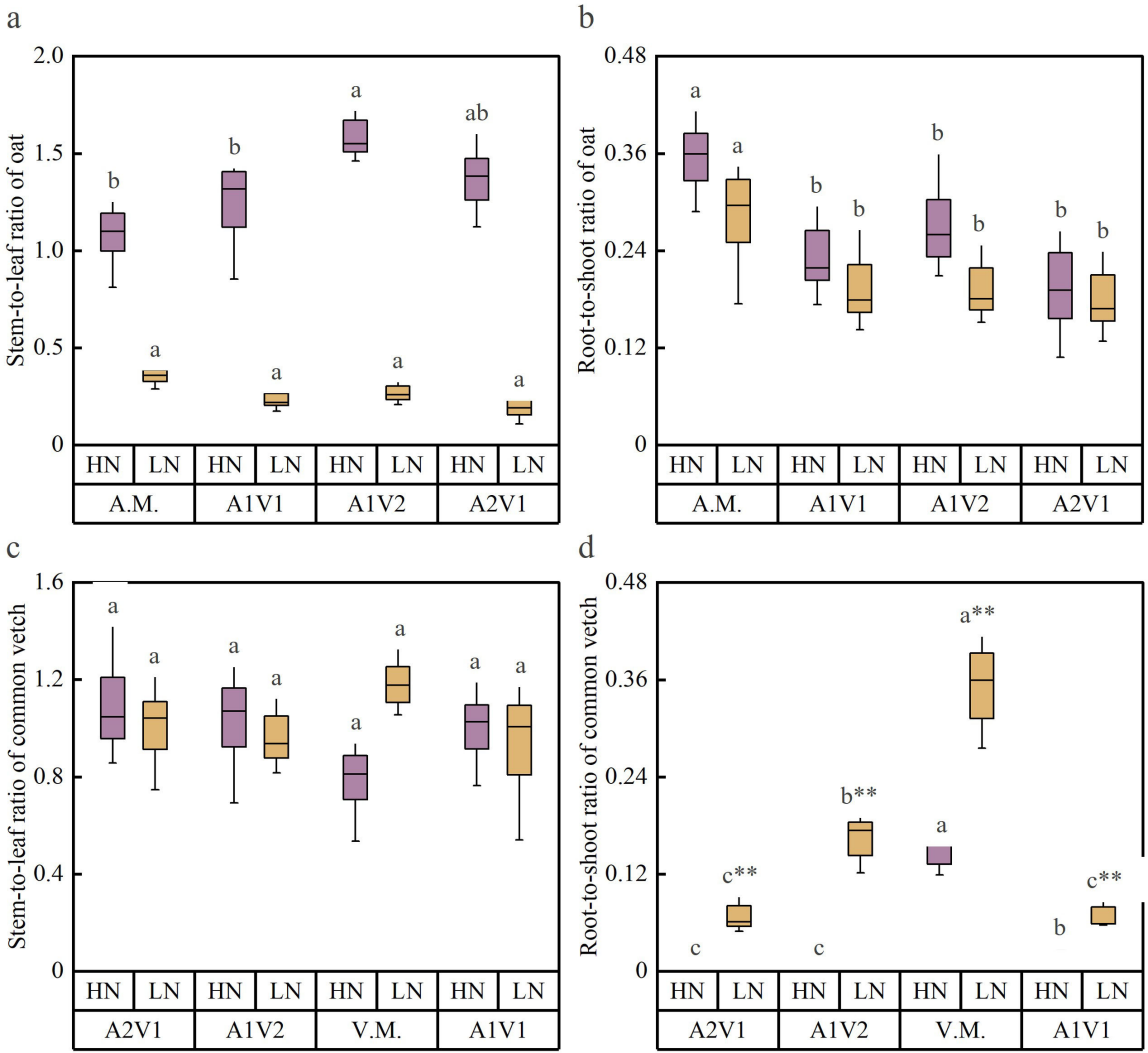
Effects of mixed sowing ratios under two N conditions on the stem-to-leaf ratio and root-to-shoot ratio of oat and common vetch. **(a)** Stem-to-leaf ratio of oat. **(b)** Root-to-shoot ratio of oat. **(c)** Stem-to-leaf ratio of common vetch. **(d)** Root-to-shoot ratio of common vetch. Different lowercase letters a, b, c indicate significant differences among different sowing ratios within the same N regime (*p* < 0.05). Superscript “*” and “**” indicate significant differences between N regimes for the same mixed sowing treatment at the 0.05 and 0.01 levels, respectively. A.M., A2V1, A1V1, A1V2, and V.M. denote different mixed sowing ratios. HN indicates N-sufficient conditions; LN indicates N-deficient conditions.

Mixed sowing ratios had a stronger effect on the stem-to-leaf ratio and root-to-shoot ratio of oat than that common vetch (**Figure 2**). The stem-to-leaf ratio of oat (**Figure 2a**) was significantly affected under the HN conditions, exhibiting a fluctuating increase with increasing oat proportion, whereas it was less affected under the LN conditions (*p* < 0.05). The root-to-shoot ratio of oat (**Figure 2b**) was also higher under HN than under LN, with the A1V1 and A1V2 treatments being significantly higher than those under LN, and in all three mixed sowing treatments the ratio increased with increasing oat proportion, with the highest value observed in oat monoculture (*p* < 0.05). For common vetch, analysis of the stem-to-leaf ratio (**Figure 2c**) and root-to-shoot ratio (**Figure 2d**) indicated that, except for the V.M. treatment, where the stem-to-leaf ratio under HN was significantly lower than that under LN (*p* < 0.05), no significant changes in stem-to-leaf ratio were observed among the other treatments (*p* > 0.05). In contrast, the root-to-shoot ratio decreased with decreasing common vetch proportion and was consistently and significantly higher under LN than under HN (*p* < 0.05).

### 3.2 Nitrogen content and accumulation in oat and common vetch mixed sowing systems plants

**Figure 3:**
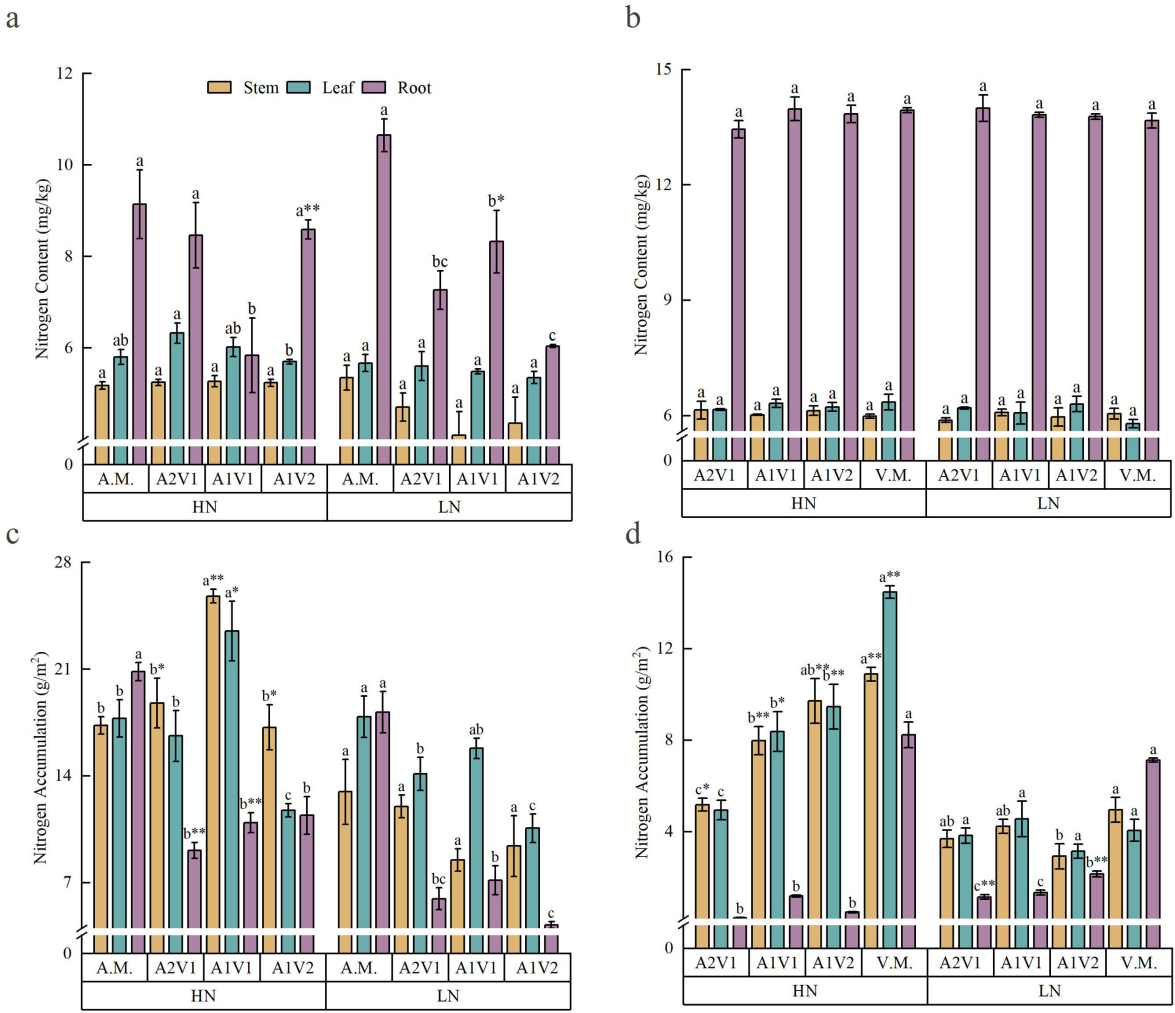
Effects of mixed sowing ratios under two nitrogen conditions on nitrogen content and nitrogen accumulation in various organs of oats and common vetch. **(a, b)** The effects of mixing ratio on nitrogen content in leaves, stems, and roots of oats and common vetch under two nitrogen environments. **(c, d)** The effects of mixing ratio on nitrogen accumulation in leaves, stems, and roots of oats and common vetch under the two nitrogen environments, respectively. Different lowercase letters a, b, c indicate significant differences among different sowing ratios within the same nitrogen regime (*p* < 0.05). Superscript “*” and “**” indicate significant differences between nitrogen regimes for the same mixed sowing treatment at the 0.05 and 0.01 levels, respectively. A.M., A2V1, A1V1, A1V2, and V.M. denote different mixed sowing ratios. HN indicates nitrogen-sufficient conditions; LN indicates nitrogen-deficient conditions.

Mixed sowing ratios and N application rates had relatively minor effects on N content in different organs of oat and common vetch, but exerted significant effects on N accumulation (**Figure 3**). As shown in **Figures 3a, b**, mixed sowing ratios only affected root N content of oat under both N regimes, with no significant effects on leaf or stem N content. Meanwhile, N content in all organs of common vetch was not significantly affected by mixed sowing ratios (*p* > 0.05). As shown in **Figure 3c**, N accumulation in oat leaves, stems, and roots was significantly higher under HN than under LN conditions (*p* < 0.05). Under both HN and LN, N accumulation in oat leaves and stems reached the highest values in the A1V1 treatment and followed an inverted “V” pattern, whereas under LN conditions it showed an overall decreasing trend. Root N accumulation decreased under both N regimes. Leaf N accumulation in oat was highest in the A1V1 treatment and lowest in the A1V2 treatment under HN conditions, while under LN conditions it decreased with the reduction in oat proportion (*p* < 0.05). In addition, root N accumulation in oat was highest in the monoculture treatment under both N regimes. Under HN conditions, root N accumulation in mixed treatments increased with increasing oat proportion, whereas under LN conditions it was relatively higher in the A1V1 treatment. As shown in **Figure 3d**, under both N conditions, N accumulation in the leaves, stems, and roots of common vetch was highest in the monoculture treatment. Under HN conditions, N accumulation in common vetch leaves and stems showed an increasing trend as the proportion of common vetch decreased in the mixture (*p* < 0.05), while under LN conditions the changes were not significant (*p* > 0.05). Root N accumulation in common vetch exhibited an overall increasing trend under both N regimes (*p* < 0.05).

### 3.3 System productivity and nitrogen use efficiency

**Figure 4:**
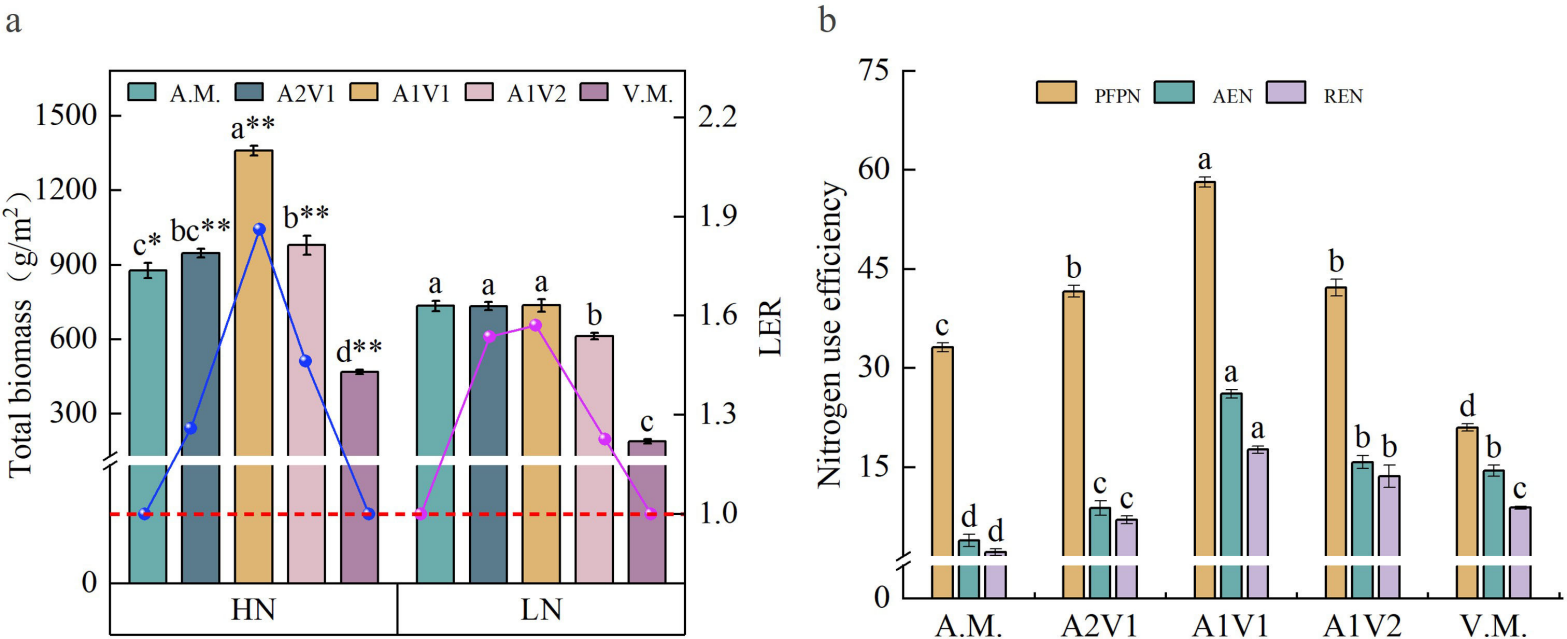
Productivity **(a)** and N use efficiency **(b)** of oat and common vetch mixed sowing systems. PFPN denotes N fertiliser productivity. AEN represents agronomic efficiency. REN signifies apparent efficiency. Different lowercase letters a, b, c indicate significant differences among different sowing ratios within the same N regime (*p* < 0.05). Superscript “*” and “**” indicate significant differences between N regimes for the same mixed sowing treatment at the 0.05 and 0.01 levels, respectively. A.M., A2V1, A1V1, A1V2, and V.M. denote different mixed sowing ratios. HN indicates N-sufficient conditions; LN indicates N-deficient conditions.

The optimisation of the plant community was ultimately reflected in system output (**Figure 4**). Under the HN conditions, the total biomass and land equivalent ratio (LER) of the A1V1 treatment were significantly higher than those of the other treatments (*P* < 0.05), reaching 1,359.81 g·m^-2^ and 1.86, respectively. In contrast, the lowest total biomass under HN occurred in the common vetch monoculture (469.26 g·m^-2^), and the LER values of both monocultures were the lowest, being equal to 1. Under the LN conditions, the total biomass was likewise lowest in the common vetch monoculture (189.78 g·m^-2^), while differences among the other treatments were relatively small (**Figure 4a**). Moreover, for the same mixture treatments, total biomass under HN was significantly higher than that under LN. These results indicate that, compared with monoculture, the HN-A1V1 treatment could save approximately 46% of the land area, reflecting exceptionally high land-use efficiency. At the same time, the N partial factor productivity, agronomic efficiency of N, and relative efficiency of N of this treatment were all significantly higher than those of the other treatments (**Figure 4b**). Specifically, the N partial factor productivity increased fivefold compared with oat monoculture and by 80.37% compared with common vetch monoculture, while REN increased sevenfold compared with oat monoculture and by 99.44% compared with vetch monoculture. This demonstrates that, while reducing N fertiliser inputs, this model achieved the maximum output per unit of N applied.

### 3.4 Comprehensive evaluation of different nitrogen conditions and mixed sowing ratios

**Figure 5:**
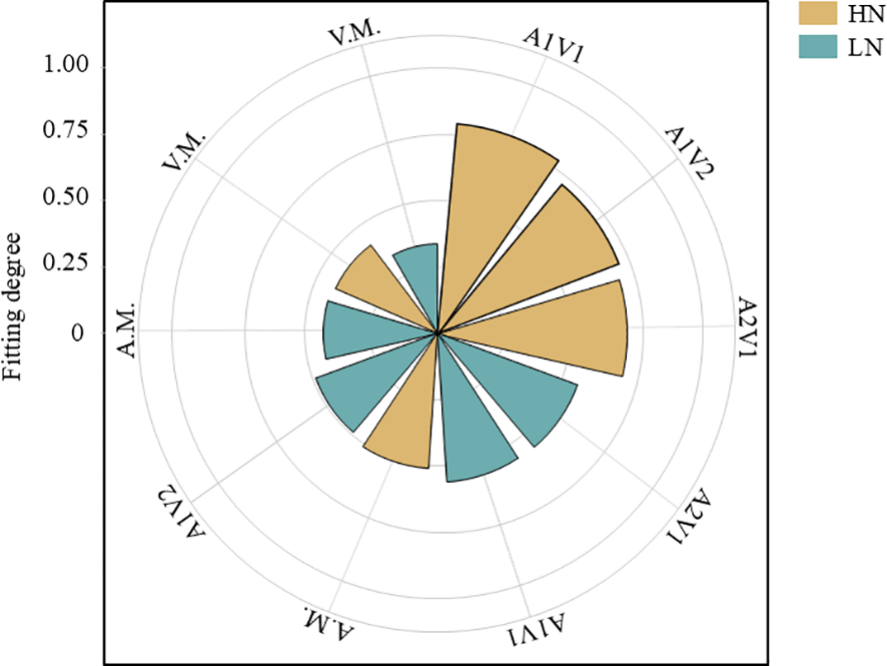
Comprehensive evaluation of oat and common vetch mixed sowing ratios under two N conditions. A.M., A2V1, A1V1, A1V2, and V.M. denote different mixed sowing ratios. HN indicates N-sufficient conditions; LN indicates N-deficient conditions.

Results indicated that under the HN conditions (**Figure 5**), the A1V1 treatment yielded the highest conformity index (0.80), whereas under the LN conditions, the V.M. treatment exhibited the lowest conformity index (0.34). Consequently, the A1V1 treatment scheme under the HN conditions represents the optimal N fertiliser level and mixed sowing ratio for this cultivation region.

The 2021 field experiment showed that oat–common vetch mixtures generally yielded more than the corresponding monocultures. The 1:1 mixture gave the most consistent advantages in forage yield, land equivalent ratio, and N uptake at both N levels. This suggests that the 1:1 mixture is a promising option for forage production on the Qinghai–Tibetan Plateau. However, the field data alone could not explain the mechanisms behind this yield advantage, especially N transfer from common vetch to oat and its response to nutrient supply. Therefore, in 2023 we conducted a ^15^N tracer pot experiment with the 1:1 mixture under a range of N and P fertilization treatments. The mechanistic results are presented below.

### 3.5 Forage yield, nutrient allocation and nitrogen use efficiency

Based on field results indicating that the 1:1 oat–common vetch mixture had the most stable yield advantage, we conducted a ^15^N tracer pot experiment. This experiment tested whether the field patterns were robust under controlled conditions and quantified N transfer between the two species under different N and P fertilization treatments. In doing so, it linked interspecific N transfer processes to the performance of the 1:1 mixture.

**Figure 6:**
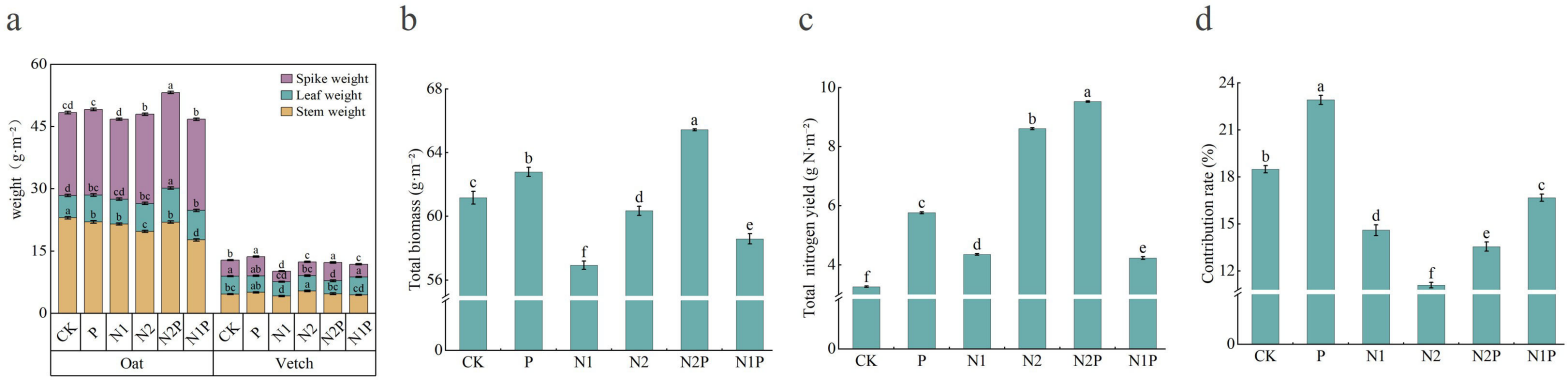
Effects of nutrient addition on forage yield and allocation in oat and common vetch mixed sowing grasslands. **(a, b)** The units for biomass of each plant organ and total biomass are g·m^-2^. **(c)** The unit for biomass N yield is g N·m^-2^. **(d)** The unit for the contribution rate of biological N fixation is %.

This study conducted one-way ANOVA followed by Tukey’s HSD *post-hoc* multiple comparisons on plant samples from six fertilization treatments (CK, P, N1, N2, N2P, N1P) to assess the effects of different fertilization regimes on stem weight, leaf weight, spike weight, total N yield, total biomass, and the contribution rate of biological N fixation. The results (**Figure 6**) showed significant differences among treatments in their effects on plant growth indicators. For oat, stem weight was highest under CK, whereas leaf and spike weights were most pronounced under combined N–P application (N2P); for common vetch, stem, leaf, and spike weights were highest under N2, CK and N1P, P and N2P treatments, respectively (*p* < 0.05). Under N-sufficient conditions, both the N2P and N2 treatments significantly increased total N yield and total biomass, and both were significantly higher than the other treatment groups (*p* < 0.05). There were significant differences among treatments in total N contribution (*p* < 0.05). Although N2P and N2 significantly enhanced total N yield, they exhibited lower contribution rates of biological N fixation, indicating reduced N allocation efficiency. The P treatment performed well in both total N yield and the contribution rate of biological N fixation, suggesting a balanced optimization of N production and utilization. The CK and N1P treatments had lower total N yield but higher contribution rates of biological N fixation, implying greater N use efficiency under lower N availability. The N1 treatment showed intermediate performance without distinct advantages.

**Figure 7:**
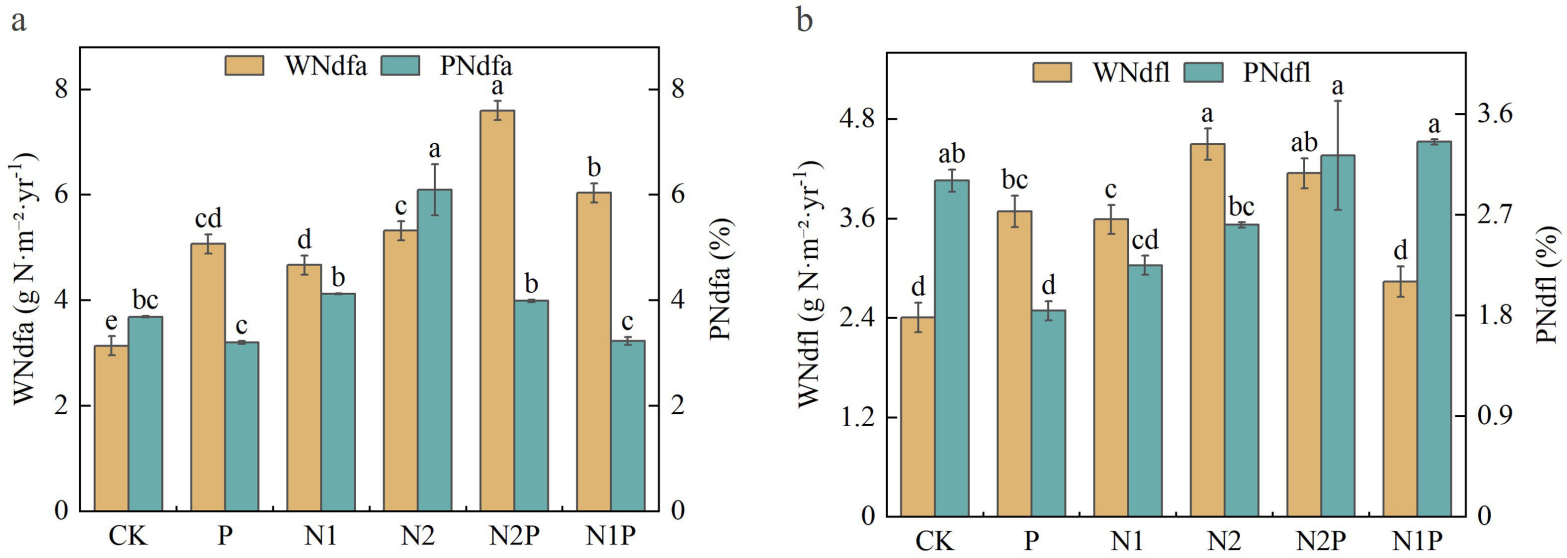
Analysis of N utilization characteristics in oat-common vetch mixed sowing grasslands. **(a)** represents N fixation and N fixation rates. **(b)** represents transfer amounts and transfer rates.

In this study (**Figure 7**), we measured the biological N fixation rate (*P*_Ndfa_), N transfer rate (*P*_Ndfl_), amount of biologically fixed N (W_Ndfa_), and amount of transferred N (W_Ndfl_) in plant samples. One-way ANOVA followed by Tukey HSD *post-hoc* test revealed significant differences among treatments (*p* < 0.05). The P treatment exhibited the lowest values in both *P*_Ndfa_ and *P*_Ndfl_, while N1 had the highest *P*_Ndfa_ but not the highest *P*_Ndfl_. CK showed moderate performance, and N1 and N1P had balanced *P*_Ndfa_ and *P*_Ndfl_. For W_Ndfa_, the CK treatment had the lowest mean value (3.14), and N2P had the highest (7.60), indicating significant differences (*p* < 0.05). For W_Ndfl_, P and N1 treatments had the highest values (3.69 and 3.59), while CK had the lowest (2.41), showing significant differences (*p* < 0.05).

**Figure 8:**
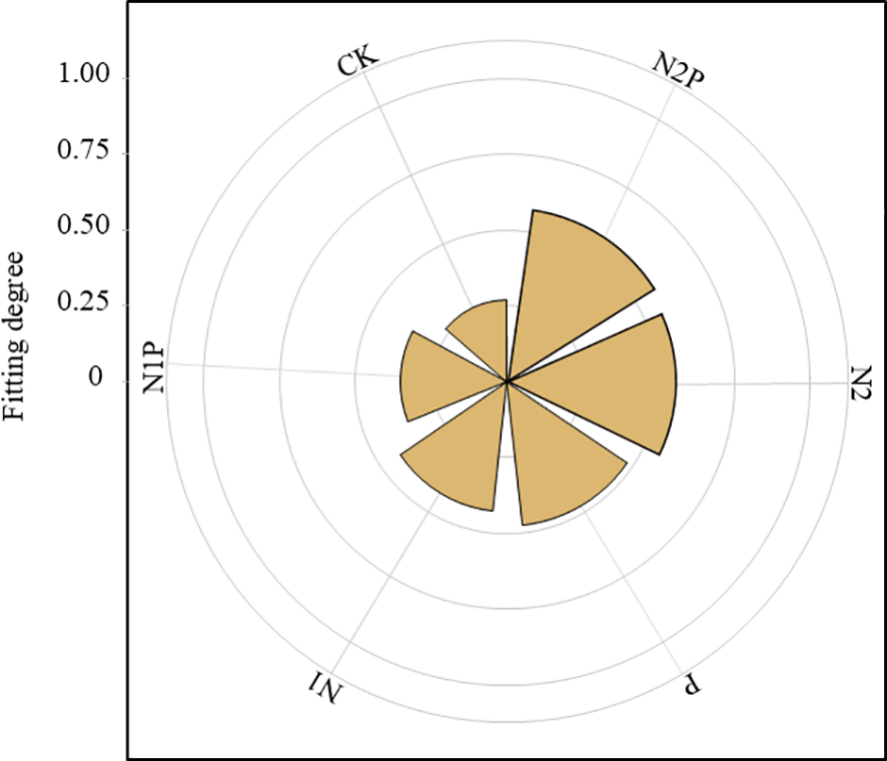
Comprehensive evaluation of N allocation in mixed-sowing grasslands.

To determine the optimal nutrient supplementation scheme, the TOPSIS multi-criteria decision model was employed to comprehensively evaluate the following parameters under different nutrient treatments: total N yield, total biomass, N fixation rate, N fixation amount, N conversion rate, N conversion amount, stem weight, leaf weight, spike weight, and biological N fixation contribution rate (**Figure 8**). Results indicated that the order of conformity from highest to lowest was N2P, N2, P, N1, N1P, and CK. Among these, N2P exhibited the highest conformity at 0.57, while CK demonstrated the lowest at 0.27. Consequently, N-P synergistic effects (N2P) significantly enhance productivity in the mixed sowing system.

**Figure 9:**
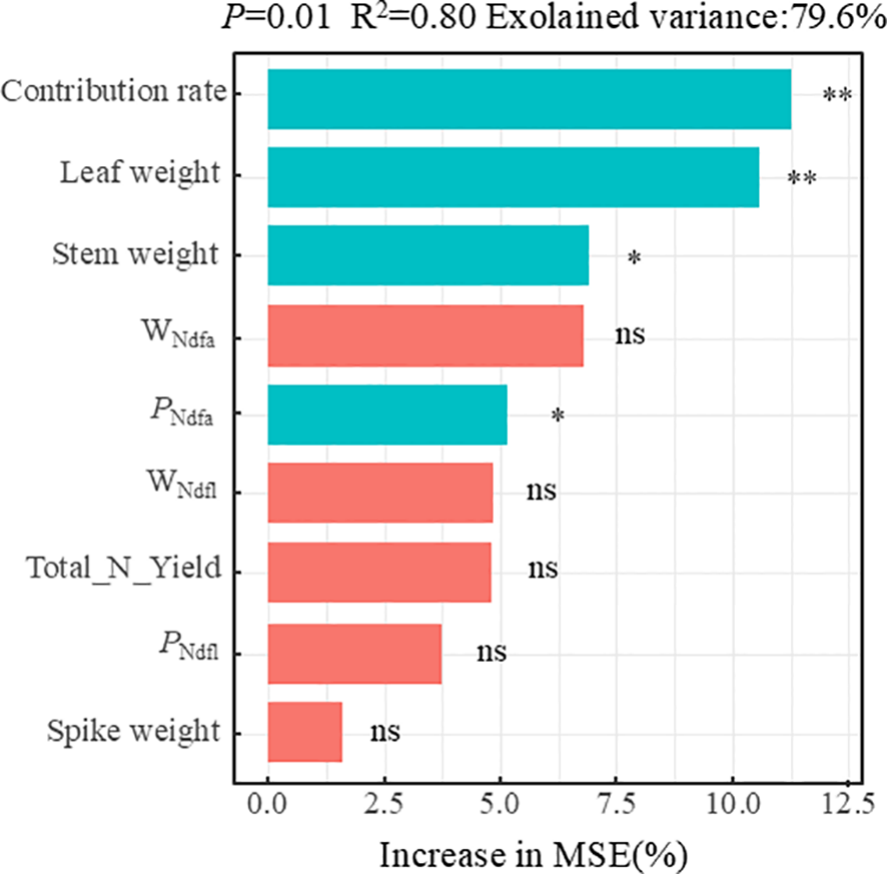
Ranking of variable importance factors for yield in mixed sowing grassland.

To determine the effects of crop agronomic traits and biological N fixation on mixed grassland yield, a random forest model was employed to rank the importance of each factor. Results indicated (**Figure 9**) that these factors collectively explained 79.6% of the variation in mixed grassland yield. Leaf weight and biological N fixation contribution rate exerted extremely significant effects on yield (*p* < 0.01), while stem weight and *P*_ndfa_ exerted significant effects (*p* < 0.05).

**Figure 10:**
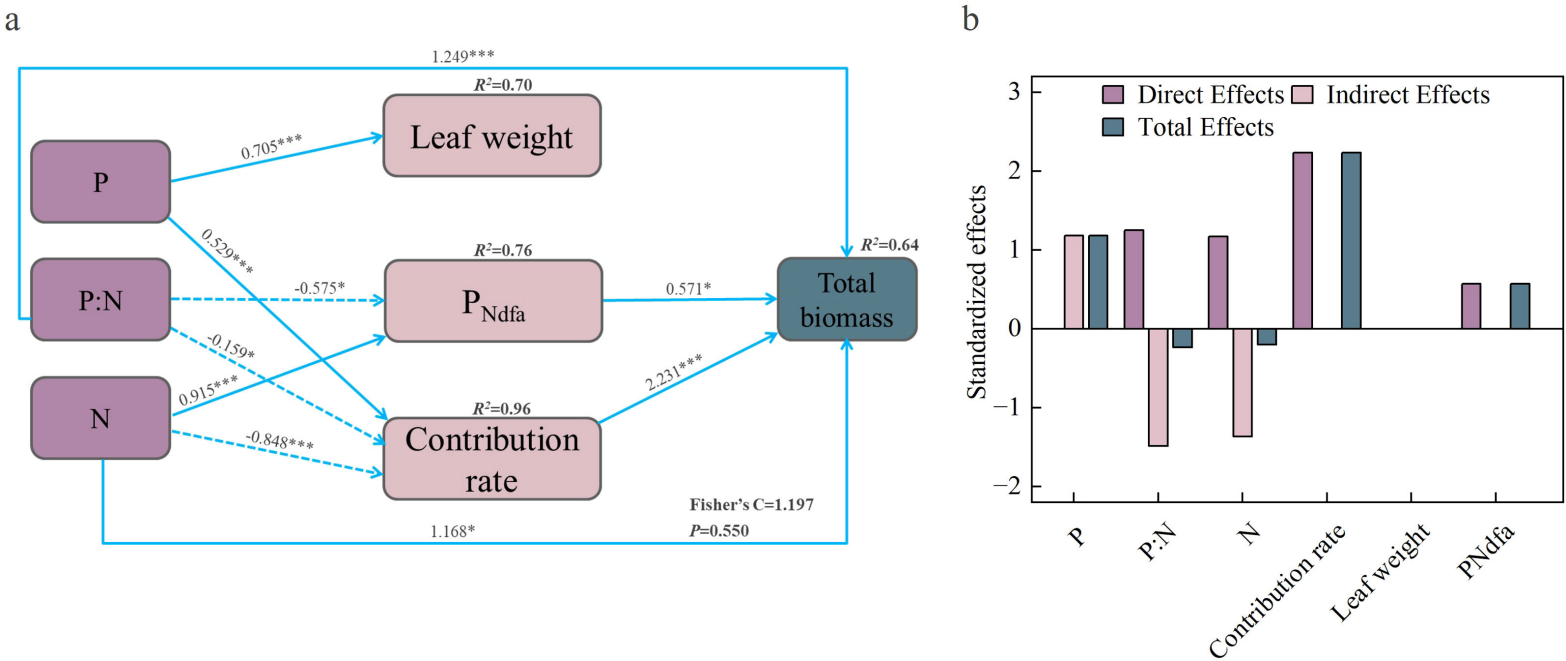
Segmented structural equation modelling analysis of the process and pathways by which nutrient addition affects total biomass in mixed sowing grasslands **(a)**, and standardised effect values for each influencing factors **(b)**.

Based on the results of the random forest model, we selected the factors that had significant effects on the yield of the mixed grassland to further construct a piecewise structural equation model and to explore the processes and pathways through which N addition, P addition, and their interaction influence mixed pasture yield (**Figure 10a**). The model showed a good fit (*p* = 0.550, Fisher’s C = 1.197). N addition and their interaction all exerted significant direct effects on mixed pasture yield, among which their interaction had the largest path coefficient (1.249), reaching a highly significant level. In addition, N addition, P addition, and their interaction indirectly affected mixed pasture yield by altering contribution rate and *P*_ndfa_. Contribution rate and *P*_ndfa_ each had direct effects on mixed pasture yield, with contribution rate showing the strongest direct effect (2.231). Summing the indirect and direct effects to obtain the total effects revealed that contribution rate had the greatest overall impact on mixed pasture yield, with a total effect of 2.231 (**Figure 10b**). Therefore, N addition, P addition, and their interaction exert direct effects on the yield of the mixed pasture. Moreover, N addition, P addition, and their interaction primarily influence mixed pasture yield indirectly through their effects on contribution rate.

## 4 Discussion

This study examined the effects of nutrient addition and sowing ratio on oat–common vetch mixed grasslands from the perspectives of productivity and N use, and used stable isotope techniques to investigate the regulation of biological N fixation and N transfer. The results demonstrated that an appropriate sowing ratios combined with N–P fertilization substantially enhanced system productivity and N use efficiency, whereas excessive N input partially suppressed N fixation by the legume component and thus undermined system sustainability.

### 4.1 Effects of sowing ratio and nutrient addition on productivity

Overall, the oat–common vetch mixture showed a clear yield advantage over the corresponding sole crops in most treatments, which is consistent with previous findings from various grass–legume intercropping or mixed sowing systems (Karpenstein-Machan and Stuelpnagel, 2000; Hu et al., 2016; Wang et al., 2019). Although mixing generally reduced the aboveground biomass of the legume within the community, oat exhibited a stronger competitive ability under mixed sowing, accumulated more dry matter and thereby increased total system yield. This is in line with Su et al (Su, 2023), who reported similar patterns in an oat–common vetch mixture. These results support the notion that yield advantages in mixed systems are largely associated with improved survival conditions, nutrient acquisition and environmental adaptation of specific component species (Yang et al., 2019), and that the performance of the dominant species often plays a decisive role in determining total system yield.

Sowing ratios affect the yield of mixed sowing systems by regulating forage biomass allocation. Sowing ratios markedly affected patterns of biomass accumulation and allocation (Li et al., 2020; Wu et al., 2020). In the present study, when oat and common vetch were sown at a 1:1 ratio (A1V1), aboveground, belowground and total biomass all reached their maximum values, and the land equivalent ratio (LER = 1.86) was significantly higher than at other ratios. This indicates a clear advantage of this configuration in terms of land use efficiency and mixed sowing benefit. Under A1V1, oat showed the highest dry matter accumulation, and the relatively large root biomass suggests a strong nutrient acquisition capacity (Hauggaard-Nielsen and Jensen, 2001; Poorter et al., 2012). Together, these findings suggest that the 1:1 sowing ratio achieves effective resource complementarity in both light capture and soil nutrient uptake, thereby supporting maximum system biomass (Lamb et al., 2011). Notably, when the oat proportion decreased from 100% to 50%, the aboveground biomass of both oat and common vetch increased. This indicates that, within the range tested, moderately increasing the legume proportion does not compromise the yield of the cereal. Instead, it improves interspecific complementarity and enhances overall system productivity, highlighting the structural and functional advantages of the 1:1 mixture (Cao et al., 2025).

Nutrient addition further amplified the yield response of the mixed system. Studies by Neumann et al (Neumann et al., 2007) and Zhao et al (Zhao et al., 2020) have shown that the sowing ratio, under N application, enhances grain yield by improving light use efficiency in cereal crops. In the pot experiment, all fertilized treatments significantly increased total biomass compared with the CK, with the N2P treatment showing the highest total and panicle biomass. This confirms that synergistic N-P supply can substantially enhance system productivity, consistent with Hauggaard-Nielsen et al (Hauggaard-Nielsen and Jensen, 2001). However, previous studies have shown that excessive N application can cause chlorophyll loss, reduce photosynthetic capacity and suppress plant growth (Liang et al., 2024), as well as increase environmental risks. In this study, the locally recommended nitrogen rate (e.g., 20 g m^-2^) effectively improved yield under the conditions tested, but from a sustainability perspective, relying solely on high N inputs to increase production is not advisable.

### 4.2 Effects of sowing ratios and nutrient addition on nitrogen acquisition and use (with isotope evidence)

Nitrogen is a key nutrient limiting productivity in alpine grasslands and shaping competitive interactions in mixed systems (Karpenstein-Machan and Stuelpnagel, 2000; Tahir et al., 2022). In typical grass-legume mixtures, grasses primarily rely on soil mineral N, whereas legumes obtain N mainly through symbiotic N fixation (Tahir et al., 2022). In this study, stable isotope techniques were used to quantify N fixation, the proportion of N derived from fixation, and N transfer from the legume to oat under different treatments, thereby elucidating the N-based mechanisms underlying the yield advantage of the mixture. The results showed that under mixed sowing conditions, particularly in the A1V1 treatment, both the amount of N fixed by the legume and the proportion of N derived from biological fixation remained relatively high, while N transfer rates and amounts from common vetch to oat increased significantly. This indicates the formation of a complementary N use pattern in the mixed system, characterized by “N fixation by the legume – partial N transfer to oat – efficient utilization by the cereal” (Jensen, 1991; Zheng et al., 2016). Through N fixation, the legume becomes partially decoupled from direct competition for soil mineral N, while oat uses both soil N and N transferred from the legume, resulting in spatial and source differentiation of N acquisition between the two species (Chalk and Smith, 2017). This niche differentiation mechanism helps explain why, under moderate N inputs, the mixed system can maintain high yield and N accumulation without extremely high external N supply.

Nutrient addition substantially altered the system N pool and related processes. The N2P treatment produced the highest total plant N yield and system N accumulation, indicating strong N uptake capacity under sufficient N and P supply (Wang et al., 2022). However, isotope data also showed that although total N fixation was highest under N2P, the proportion of N derived from fixation was relatively low, consistent with the general pattern that high N availability suppresses symbiotic N fixation in legumes (Abd-Alla et al., 2023). In contrast, the P-only treatment showed a more balanced performance in both N fixation rate and amount, suggesting that under relatively low external N inputs, P fertilization can significantly promote legume N fixation and enhance internal N supply to the system (Cui et al., 2022). N transfer and N use efficiency also differed markedly among fertilization regimes. N2P exhibited the highest N transfer rates and amounts, indicating that N-P co-application can enhance N transfer from the legume to oat and thereby further improve system productivity (Jensen, 1991). However, N1P showed superior performance in both N transfer and N use efficiency, implying that moderate N-P supply achieves a better balance between maintaining high N fixation and enhancing N use efficiency. Although N concentrations in individual organs or whole plants did not differ consistently among treatments, mixed sowing generally resulted in higher aboveground biomass and N accumulation, indicating that, at the system scale, the mixture improved both N uptake and utilization (Zhang and Li, 2003).

Sowing ratio also significantly influenced N acquisition and use by altering interspecific competition and root distribution (Li et al., 2003; Zhu et al., 2018). In the A1V1 treatment, root biomass was highest, and both system N accumulation and the contribution of N fixation were relatively high, suggesting that this ratio favors a pattern of “root complementarity – N source complementarity”. As oat proportion decreased from 100% to 50%, the aboveground biomass of both oat and common vetch increased, indicating that, within the tested range, moderately increasing the legume proportion did not reduce N acquisition by the cereal. Instead, it enhanced complementarity and improved N use efficiency of the entire system (Liang et al., 2024; Wu et al., 2022).

### 4.3 Implications for fertilization and mixed sowing strategies in alpine grasslands

Taken together, the results on productivity and N use have important implications for managing oat–common vetch mixed grasslands in alpine regions. First, compared with traditional practices relying on high N inputs, optimizing the sowing ratio (e.g., a 1:1 oat–common vetch mixture) can substantially increase system yield and land use efficiency without greatly increasing external inputs (Hou et al., 2025). Second, moderate N-P fertilization helps to simultaneously enhance yield, N use efficiency and legume N fixation (Yu et al., 2018). High N-P input (e.g., N2P) may be appropriate where the primary goal is to maximize yield and total N accumulation, whereas moderate N-P input (e.g., N1P) or P-only application is more suitable for balancing productivity, N use efficiency and N fixation, thereby reducing dependence on external N inputs and lowering environmental risks.

The isotope results further indicate that the N advantage of mixed systems primarily arises from optimized N fixation and N transfer processes (Luo et al., 2024; Barneze et al., 2025). Future management strategies and research should therefore pay greater attention to rhizobial activity, temporal dynamics of N fixation, and N transfer under different fertilization regimes and sowing ratios, as well as to the roles of rhizosphere microorganisms and root exudates in regulating these processes. Overall, this study demonstrates that, through the combination of a rational sowing ratio, moderate nutrient addition, and isotope-based process analysis, it is possible to achieve a coordinated improvement in productivity and N use efficiency in alpine mixed grasslands, thereby providing a scientific basis for developing high-yield, resource-efficient and environmentally friendly grassland production systems in alpine regions.

## 5 Conclusion

This study demonstrates the significant impact of N treatments on growth traits, N accumulation, and N fixation efficiency in mixed sowing systems of oats and common vetch. The A1V1 treatment (1:1 ratio) showed superior performance, achieving the highest aboveground biomass (1164 g·m^-2^) and optimal N utilization with a LER of 1.86. Oats played a crucial role in enhancing growth indicators under HN conditions, contributing to overall system performance. Mixed sowing significantly improved N transfer and fixation, with the P treatment excelling in N fixation (31.86%) and transfer rates (18.44%), highlighting the role of P in enhancing rhizobial activity. Moderate N-P co-application (N2P) also improved N transfer (4.14 g·m^-2^). The success of the A1V1 treatment is attributed to balanced N allocation, enhanced competition for light, and efficient nutrient acquisition. These findings underscore the potential of rational N and P addition to promote sustainable agriculture by enhancing crop resilience, productivity, and environmental benefits.

## Data Availability

The original contributions presented in the study are included in the article/supplementary material. Further inquiries can be directed to the corresponding author.
